# Assessor discomfort and failure to fail in clinical performance assessments

**DOI:** 10.1186/s12909-023-04688-1

**Published:** 2023-11-27

**Authors:** Catherine E Scarff, Margaret Bearman, Neville Chiavaroli, Stephen Trumble

**Affiliations:** 1 Department of Medical Education, Melbourne Medical School, University of Melbourne, Room N722, Level 7 North Medical Building Grattan Street, Melbourne, VIC Australia; 2 Centre for Research in Assessment and Digital Learning (CRADLE), Deakin University, Melbourne, VIC Australia; 3 Australian Council for Educational Research, Camberwell, Australia

**Keywords:** Failure to fail, Workplace assessment, In-training assessment, Feedback, Judgement, Clinical supervisor

## Abstract

**Background:**

Assessment of trainee performance in the workplace is critical to ensuring high standards of clinical care. However, some supervisors find the task to be challenging, and may feel unable to deliver their true judgement on a trainee’s performance. They may ‘keep MUM’ (that is, keep mum about undesirable messages) and fail to fail an underperforming trainee. In this study, we explore the effect of discomfort on assessors.

**Methods:**

Using a survey method, supervisors of trainees in the Australasian College of Dermatologists were asked to self-report experiences of discomfort in various aspects of trainee workplace assessment and for their engagement in MUM behaviours including failure to fail.

**Results:**

Sixty-one responses were received from 135 eligible assessors. 12.5% of assessors self-reported they had failed to fail a trainee and 18% admitted they had grade inflated a trainee’s score on a clinical performance assessment in the previous 12-month period. Assessors who reported higher levels of discomfort in the clinical performance assessment context were significantly more likely to report previously failing to fail a trainee. The study did not reveal significant associations with assessor demographics and self-reports of discomfort or MUM behaviours.

**Conclusions:**

This study reveals the impact of assessor discomfort on the accuracy of assessment information and feedback to trainees, including as a contributing factor to the failure to fail phenomenon. Addressing assessor experience of discomfort offers one opportunity to impact on the complex and multifactorial issue that failure to fail represents.

## Background

Clinical supervisors carry heavy loads: they must assess trainees while also teaching and supporting them and ensuring safe patient care. Assessment in the workplace is integral to ensuring trainee competence [1] and tools such as Workplace Based Assessments (WBA) and In Training Assessments (ITA) are commonplace in many health professional training settings. While some supervisors do not have any concerns assessing trainees in the workplace, others do report challenges and complexity [2]. And when combined with other pressures they face, these challenges may at times impair the assessment messages they ultimately deliver to trainees. For example, the resulting message may be weakened so that it does not convey the assessor’s considered judgement.

The failure to fail literature describes a gap that sometimes may exist between assessors’ private thoughts on the trainee’s performance and the public judgement they deliver [3] with the two not always aligning [4, 5]. In the case of a poorly performing trainee in particular, an assessor may not have the time [6] nor resolve to provide their true judgement especially if they are unsure about what to document, anticipate they will be required to create a remediation plan or that the trainee will challenge their result and require the assessor to justify their decision [7]. In their systematic review, Yepes-Rios and colleagues summarise barriers to delivering negative assessment information into those which relate to supervisor and trainee considerations, the assessment tools and culture in which assessment occurs and importantly, the procedures in place around remediation for failing trainees [8]. With all these potential barriers, failure to fail may present a more attractive solution for some assessors. While the introduction and widespread implementation of competency-based medical education (CBME) has changed the assessment landscape, its impact on failure to fail is still largely unexplored, though it likely persists. For example, Chin and colleagues’ study [9] shows that social aspects and relationships still have a large impact on supervisor’s assessment practices and that discomfort with aspects of assessment is relevant.

Outside the medical education context, authors have explored how discomfort impairs the delivery of performance appraisal information in business settings. Performance appraisals have several parallels with workplace assessments in medical education, and a scale to assess rater discomfort in that context has been developed [10]. Others have subsequently explored the relationship between discomfort and the phenomenon known as the MUM effect [11] – which describes people generally preferring to avoid uncomfortable communications of unfavourable news or keep “Mum about Undesirable Messages”. MUM plays on the English expression to keep ‘mum’ or silent about something, with ‘mum’ referring to the “inarticulate sound made with closed lips, usually as an indication or inability or unwillingness to speak” [12]. In a simulated setting, Cox and colleagues saw that the higher the level of discomfort reported by those conducting an appraisal, the more likely they were to engage in MUM behaviours [13].

The MUM effect provides a lens through which to view instances where an assessor is unwilling to deliver a negative judgement on a trainee’s performance [14]. The MUM effect predicts a teller will be reluctant to deliver a message they perceive will be viewed as negative by the recipient, and instead opt to delay giving the message [11], distort (or sugarcoat) the message [15] or avoid giving it altogether [11]. The reasons for choosing to keep MUM may lie in concerns about the outcome of delivering a negative message, for either the teller or the recipient, or be due to the prevailing norms of the setting [16]. In the medical education context, keeping MUM may result in failure to fail or assigning a higher grade than is appropriate (grade inflation). The impact will vary across the assessment context, for example keeping MUM in a feedback conversation will differ from the situation of not documenting corrective feedback comments on a low stakes clinical performance assessment or not documenting negative assessment judgments on a high-stakes one. However, the missed opportunity and lack of impact on learning, mean all scenarios may potentially result in negative outcomes for the patient and the learner [17] as well as the community.

While assessor training is still regarded as a priority for addressing failure to fail [17] and ‘skill’ in assessment is undoubtedly of key importance, assessors also need the ‘will’ to deliver their judgement [7]. The MUM literature permits a means to examine the disconnect between private thoughts and public judgements. Therefore, we seek to explore assessor experiences of discomfort in the workplace assessment context in medical education, and whether such discomfort impacts on assessors’ ‘will’ to deliver their message in full, and thus the public outcomes of their assessment.

Our study is contextualised within national specialty training in dermatology in Australia, where the tensions between being a colleague and a trainee are heightened within a small community, and therefore provides a useful microcosm to study the potentially uncomfortable nature of work-based assessment. Within this setting, we compare assessors’ experiences of discomfort in with their self-reported MUM behaviours, including failure to fail. Our research questions for this study are: What are Australian dermatology assessors’ reported experiences of discomfort in relation to their assessor role? What is the relationship between their level of discomfort and self-report of MUM behaviours in clinical performance assessments?

## Methods

Overview of the study:

We administered an anonymous questionnaire to explore Australian dermatology assessors’ experiences of giving clinical performance assessment information to dermatology trainees in Australia.

### Study context

The study was set in the Australasian College of Dermatologists (ACD); challenges around discomfort, including fears and concerns, have been reported in clinical performance assessments in this context [18, 19]. As a national training program, this setting provides the opportunity to obtain a broad range of perspectives.

### Sample

Fellows of the Australasian College of Dermatologists (ACD) who self-identified as a supervisor of trainees and with recent (within the previous 12 months) experience of assessing trainees were invited to participate (n = 135). Informed consent was implied by submission of the questionnaire.

### Questionnaire development

The questionnaire included demographic questions and items from two previously published questionnaires, modified for the local context.

#### Demographic information

Demographic information collected included gender, state of practice, years as clinician and assessor, and type of assessor (clinical supervisor (CS), or supervisor of training (SoT), who is the senior supervisor at a given training site).

#### Assessor self-report of discomfort with assessment processes

The Performance Appraisal Discomfort Scale (PADS) [in [10]] was used to measure assessor discomfort. The PADS is a 20-item scale that asks respondents to rate their experience of discomfort with certain tasks in the performance appraisal setting. Examples include situations such as giving feedback to a poor performer or dealing with accusations of favouritism in providing ratings. Modifications made to the PADS included changing the terms ‘employee’ and ‘subordinate’ to ‘trainee’ and the term ‘interview’ to ‘feedback session’. A five-point Likert scale was used for assessors to rate their experience of discomfort with the tasks from ‘no discomfort’ to ‘high discomfort’.

#### Assessor self-report of MUM behaviours

Assessors who reported recent delivery of negative feedback to a trainee were presented with the MUM effect scale [20]. This is used to measure self-report of assessor engagement with MUM behaviours and the authors report validation studies in the MUM behaviours of distortion (sugarcoating) and avoidance. The original MUM effect scale included questions related to the norms of the organisation, but as these were not the focus in our study they were not included.

Three additional questions, based on the authors’ own questions and other sources [21] were added. Examples of modifications to the MUM effect scale include changing items from “I asked others to give any negative information” to “I asked another consultant to give the trainee any negative information”. The MUM effect scale also used a 5-point Likert scale where assessors reported their level of agreement or disagreement with engaging in a list of behaviours.

#### Assessor self-report of failure to fail and grade inflation

All supervisors were asked to self-report if they had avoided assigning a failing result appropriately to a trainee (failed to fail) or had given a trainee a higher rating than was warranted (grade inflated) during the previous 12 months.

### Distribution

The questionnaire was distributed electronically (SurveyMonkey, Momentive Inc. San Mateo, California, USA, www.momentive.ai) by the ACD to all Fellows and responses were collected between 12 August and 16 September 2016.

### Analysis

Results were imported into SPSS Statistics (IBM Corp. Released 2017. IBM SPSS Statistics for Windows, Version 25.0. Armonk, NY: IBM Corp.) for statistical analysis. Comparisons of responses by group were made using independent samples t-tests and inferences for proportions.

This questionnaire study formed part of a larger research project, being the PhD project for the first author [3]. Ethics approval for the study was obtained from the Ethics committee of the Department of Medical Education HEAG University of Melbourne, Ethics ID 154,565.

All methods were carried out in accordance with relevant guidelines and regulations. Informed consent from all subjects was implied by the submission of their questionnaire.

## Results

### Demographic information

The response rate to the questionnaire was 45.2% (61 responses from 135 eligible assessors) with responses received from supervisors in six of the eight Australian states and territories. 58% of respondents were male and 69% reported having been involved in assessing trainees for five or more years.

### Assessor self-report of discomfort with assessment processes

Assessors reported a wide range of discomfort with the elements of the assessment process. For all 20 items, reported levels of discomfort ranged from none to at least ‘3’ on the 5-point Likert scale, giving a mean PADS score across all items of 2.45. The item with the lowest level of discomfort (mean = 1.21) was ‘conducting a feedback session with a trainee who is performing well’ (n = 44, 83% reported ‘no discomfort’). The item with the highest level of discomfort reported (mean = 3.71) was warning a trainee that the assessor would support their removal from the training program unless performance improved (n = 14, 29% reported ‘high discomfort’). Independent t-tests revealed that the mean PADS score did not show statistically different variation with a range of assessor demographics, including gender or seniority as an assessor or clinician (Table 1).

**Table 1:** Assessor self-reported levels of discomfort in feedback giving situations across all respondents

Description of situation	Mean PADS score	Range
Conducting a feedback session with a trainee who is performing well	1.21	1–4
Letting a trainee talk during a feedback session	1.28	1–3
Letting a trainee give his or her point of view regarding a problem with performance	1.52	1–4
Giving a satisfactory rating to a trainee who has done a satisfactory (but not exceptional) job	1.52	1–4
Encouraging a trainee to evaluate his or her own performance	1.57	1–3
Telling a trainee that he or she must stop taking long breaks	2.10	1–4
Asking a trainee if he or she has any comments about your ratings of his or her performance	2.13	1–4
Telling a trainee that he or she must stop coming to work late	2.15	1–4
Talking to a trainee about his or her performance on the job	2.29	1–5
Telling a trainee that his or her performance can be improved	2.38	1–5
Telling a male trainee that his performance needs to improve	2.51	1–5
Telling a female trainee that her performance must improve	2.63	1–5
A trainee’s challenging you to justify your evaluation in the middle of a feedback session	2.84	1–5
Telling a trainee that his or her work is only satisfactory, when you know that he or she expects an above satisfactory rating	2.85	1–5
Telling a trainee who has problems in dealing with others that he or she should do something about it	2.94	1–5
Responding to a trainee who is upset over your rating of his or her performance	3.08	1–5
Conducting a feedback session with a poorly performing trainee	3.30	1–5
A trainee’s accusing you of playing favourites in the rating of trainees	3.37	1–5
Supporting a recommendation that a trainee be removed from the training program	3.54	1–5
Warning a poorly performing trainee that unless performance improves, you will support a recommendation for his or her removal from the training program	3.71	1–5

1 = ‘no discomfort’, through to 5 = ‘high discomfort’ and range of scores reported by respondents, Performance Appraisal Discomfort Scale (PADS) [10]

### Assessor self-report of MUM behaviours

Just under half the respondents reported that they had delivered some negative assessment information to a trainee in the preceding 12 months (n = 23, 46%).

This group self-reported minimal engagement in MUM behaviours except for one item “I tried to make the trainee feel better by emphasising positive things”, where 81.8% reported doing this. The mean PADS scores between those who reported sharing some negative assessment information did not differ from those who did. Similarly, self-reports of MUM behaviours did not vary significantly based on demographic features (Table 2).

**Table 2:** Assessor self-report of MUM behaviours in relation to giving negative assessment messages to trainees

Possible behaviours in relation to giving negative assessment messages to trainees	MUM behaviour	Agree n (%)	Disagree or neutral n (%)	Total number of respondents n
I tried to make the trainee feel better by emphasising positive things	SC	18 (81.8)	4 (18.2)	22
I put the negative feedback between two positive comments	SC	6 (27.3)	16 (72.7)	22
I did not point out all of the weaknesses	A	5 (21.7)	18 (78.3)	23
I tried to make it sound nicer than it was	SC	4 (19.0)	17 (81.0)	21
I adopted a very formal or clinical manner when I gave the feedback so the trainee would be less likely to question me	O	4 (19.0)	17 (81.0)	21
I told them only part of the problem	A	4 (17.4)	19 (82.6)	23
I left out some on the negative information	A	4 (17.4)	19 (82.6)	23
I tried to make the feedback more positive than it really was	SC	3 (13.6)	19 (86.4)	22
I changed the feedback or rating so it was not so negative	SC	2 (9.1)	20 (90.9)	22
I changed the feedback during the feedback session due to the trainee’s response to it	O	1 (4.8)	20 (95.2)	21
I changed the feedback or rating to make it more positive	SC	1 (4.5)	21 (95.5)	22
I didn’t go into much detail about the negatives	A	1 (4.3)	22 (95.7)	23
I asked another consultant to give the trainee any negative information	A	1 (4.3)	22 (95.7)	23
I left giving the feedback for as long as I could	A	1 (4.3)	22 (95.7)	23
I avoided the trainee at work, so I would not have to talk to him/her	A	0	23 (100)	23
I avoided it altogether	A	0	23 (100)	23
I steered clear of circumstances in which I’ll have to give negative information	A	0	23 (100)	23
I found ways to get out of sharing negative information	A	0	23 (100)	23
I gave the feedback as quickly as I could and before the trainee could react to it	O	0	21 (100)	21

MUM behaviours indicated by A – avoidance, SC – sugarcoating/distortion, O – other behaviours [19]

### Assessor self-report of failure to fail and grade inflation

**Fig. 1:**
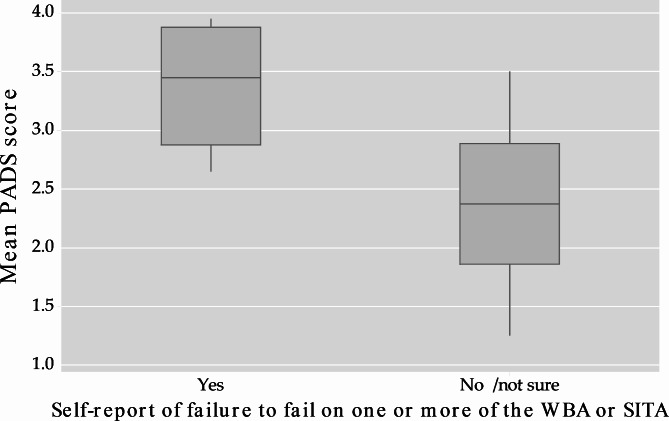
Box plot of mean PADS score compared with self-report of failure to fail on one or more WBA or Summative In-Training Assessment (SITA)

All respondents were asked to report anonymously whether they had failed to fail or grade inflated a trainee in the previous 12 months. Six (out of 48, = 12.5%) assessors self-reported they had failed to fail a trainee and eight (out of 45, = 18%) admitted they had grade inflated a trainee’s score on a clinical performance assessment in the previous 12-month period. A statistically significant relationship between PADS score and self-report of failure to fail was seen with mean PADS score of 3.38 (n = 6) compared with 2.35 (n = 40) (95% CI difference 0.512, 1.495, *p = 0.04*) (Fig. 1).

Self-report of failure to fail on WBA or an ITA and grade inflation on an ITA showed no significant relationship to seniority as a consultant, experience as an assessor, gender or SoT status. Although the difference in proportions of those failing to fail was not significantly different based on SoT status, no respondent who admitted to failure to fail was an SoT.

## Discussion

To our knowledge, this is the first study to explore supervisor experiences of feelings of discomfort in delivering negative assessment information in a clinical performance assessment context. It shows that, within the context of Australian dermatology, some assessors do self-report engaging in MUM behaviours, including failure to fail in the clinical performance assessment setting and, further a significant association between an assessor’s reported level of discomfort as measured by the PADS scale and their self-report of recent failure to fail. As assessment practices in specialist medical colleges tend to be similar in both formats and approaches, we suggest that these findings have relevance to other contexts.

While the study clearly reveals a relationship between assessor’s discomfort and self-report of failure to fail, a wide inter-individual variation was seen, with some assessors reporting no discomfort for situations that caused others high levels. Previous studies point to this being a cause of concern when the determination of competence relies primarily on assessors’ judgements. Friedman and colleagues have also noted the persisting issue of failure to fail as a risk for the move towards CBME, with its requirement for supervisors of trainees to assess their trainees and accurately report their findings [17]. In their empirical study of a simulated trainee performance, they report 17.7% of assessors failed to fail a trainee whose performance was clearly unsatisfactory (as determined by expert opinion). This was despite the trainee being unknown to the assessor, and there being no requirement to deliver the result to them directly [17] – factors which may encourage assessors to deliver the truth.

Similarly, in the nursing context, Hauge and colleagues report that 16.8% of nurses admitted to previously failing to fail a nursing trainee [22]. In our study, 12.5% of assessors admitted to failing to fail a trainee within the preceding 12 months. With the increasing move to CBME and reliance upon the judgments of assessors who work alongside the trainee they are assessing, these figures are concerning. It is not known what contribution discomfort with the assessment process made to the other studies but, as shown by our work, those assessors who report more discomfort also report a greater likelihood of failing to fail. Therefore, alongside strategies recommended to reduce failure to fail such as improved training in educational methods including documentation [22] and assessment [17] and impressing upon assessors their professional and ethical obligations [23], efforts should also further explore the impact of discomfort on reported assessment outcomes. While broadening the sample of assessors who provide input on trainee performance may partly mitigate this situation, there are further aspects to address. We agree with Hughes and colleagues on the urgent need to embark on “further quality research specifically exploring the impact that moral distress may have on failure to fail” [23]. We argue for exploring discomfort as a predictor of failure to fail, including whether it is fixed or variable and how it may be influenced by factors such as assessor training.

We also propose that assessor discomfort be addressed in relation to the impact that the assessor roles has on some clinicians and their assessments. The PADS scale, modified for local use, may have a role identifying clinicians who are vulnerable to high levels of discomfort in assessment and further study could explore whether PADS scores are modifiable with variables such as training or experience. For example, Cox and colleagues suggested role play for those with high levels of discomfort [13]. Assessor discomfort certainly does not explain all instances of failure to fail, but it does represent a further avenue to pursue to both ensure that assessors are not overburdened by their responsibilities and trainees are provided with the accurate assessment information they need and deserve.

Failure to fail and grade inflation have been well documented in medical education settings [24–26] and have potentially severe consequences. With the increasing assessment of trainees in the workplace and moves to programs of assessment, ensuring that assessment judgments and feedback conversations aimed at learner development are not impeded by assessor discomfort is important. CMBE and programmatic models of assessment depend on comments from WBA fulfilling a double duty: to provide material for summative decisions and to promote learning and development [27]. Failure to deliver accurate assessment information therefore undermines an important purpose of these assessments.

Failure to fail is of concern on many levels, not least of which are risks to patient safety and damage to the reputation of the profession. Authors have suggested many possible targets in the effort to reduce failure to fail in medical education. This study adds to the literature by showing the relationship between assessor discomfort and self-report of failure to fail, thus presents a new opportunity for further study and intervention, both in relation to the impact of assessor discomfort on the assessment result delivered and the further regarding the impact it may have on the assessors themselves.

### Limitations

There are several limitations to this study. We conducted our research in a particular context, the assessments associated with Australasian College of Dermatologists. The questionnaire response rate of 45.2%, whilst considered satisfactory for an electronic survey and in keeping with response rates from other surveys [28], still meant many possible responders did not contribute their experiences. Questionnaires are subject to various biases, including responder bias, whereby those with more extreme views on a subject may be more likely to respond. We attempted to minimise this effect by surveying the whole assessor population, rather than a sample. All parts of the questionnaire relied on self-report of behaviours, and no independent measures were included. This introduces potential inaccuracies to the results, including the social desirability bias whereby people are more likely to give socially acceptable responses. This study also focused on the individual supervisor rather than considering the system in which they work which may lead to or promote certain behaviours. Finally, the quantitative data presented here can give information as to the scope of issues, but not explore the reasons behind behaviours. This is important to pursue to further understand the issues and additional studies are planned.

## Conclusions

Assessors of medical speciality trainees have many roles and responsibilities. Many experience high levels of discomfort particularly in relation to delivering negative assessment information to trainees and this study shows that MUM behaviours, including failure to fail and grade inflation can and do occur in medical specialty training settings. Failure to fail is a multifactorial and complex phenomenon which requires a multifactorial solution. Assessor discomfort clearly impacts the message they deliver to the trainee and needs to be further explored and addressed for the sake of the assessor experiencing it, the trainee, whose assessment results may potentially be affected by it, and the patients whose care may ultimately be below the required standard.

## Data Availability

The datasets used and/or analysed during the current study are available from the corresponding author on reasonable request.
